# Patient Management Strategies and Long-Term Outcomes in Isolated Distal Deep-Vein Thrombosis versus Proximal Deep-Vein Thrombosis: Findings from XALIA

**DOI:** 10.1055/s-0039-1683968

**Published:** 2019-03-26

**Authors:** Walter Ageno, Lorenzo G. Mantovani, Sylvia Haas, Reinhold Kreutz, Danja Monje, Jonas Schneider, Jörg-Peter Bugge, Martin Gebel, Alexander G. G. Turpie

**Affiliations:** 1 Department of Medicine and Surgery, University of Insubria, Varese, Italy; 2 CESP-Center for Public Health Research, University of Milano-Bicocca, Monza, Italy; 3 Formerly Institute for Experimental Oncology and Therapy Research, Technical University of Munich, Munich, Germany; 4 Charité-Universitätsmedizin Berlin, corporate member of Freie Universität Berlin, Humboldt-Universität zu Berlin, and Berlin Institute of Health, Institute of Clinical Pharmacology and Toxicology, Berlin, Germany; 5 Bayer AG, Berlin, Germany; 6 Bayer AG, Wuppertal, Germany; 7 Department of Medicine, Hamilton Health Sciences, Hamilton, Ontario, Canada

**Keywords:** deep-vein thrombosis, rivaroxaban, routine clinical practice, venous thromboembolism

## Abstract

**Background** Overall, 30 to 50% of lower-limb deep-vein thrombosis (DVT) cases are isolated distal DVT (IDDVT). The recurrent venous thromboembolism (VTE) risk is unclear, leaving uncertainty over optimal IDDVT treatment. We present data on patients with IDDVT and proximal DVT (PDVT) from the prospective, noninterventional XALIA study of rivaroxaban for acute and extended VTE treatment.

**Methods** Patients aged ≥18 years scheduled to receive ≥3 months' anticoagulation with rivaroxaban or standard anticoagulation were eligible, with follow-up for ≥12 months. We describe baseline characteristics, management strategies, and incidence proportions of VTE recurrence, major bleeding, and all-cause mortality in patients with IDDVT or PDVT, with or without distal vein involvement.

**Findings** Overall, 1,004 patients with IDDVT and 3,098 with PDVT were enrolled; 641 (63.8%) and 1,683 (54.3%) received rivaroxaban, respectively. Patients with IDDVT were younger and had lower incidences of renal impairment, cancer, and unprovoked VTE than those with PDVT. On-treatment recurrence incidences for IDDVT versus PDVT were 1.0 versus 2.4% (adjusted hazard ratio [HR]: 0.56; 95% confidence interval [CI]: 0.29–1.08), and incidences posttreatment cessation were 1.1 versus 2.1% (adjusted HR: 0.65; 95% CI: 0.32–1.35), respectively. On-treatment major bleeding incidences were 0.9 versus 1.4% and mortality was 0.8 versus 2.2%, respectively. Median treatment duration in patients with IDDVT was shorter than in those with PDVT (102 vs. 192 days, respectively).

**Interpretation** Patients with IDDVT had fewer comorbidities and were more frequently treated with rivaroxaban than those with PDVT. On-treatment and posttreatment recurrences were less frequent in patients with IDDVT.

**Trial registration number:** NCT01619007.

## Introduction

Isolated distal deep-vein thrombosis (IDDVT) accounts for around 30 to 55% of all deep-vein thrombosis (DVT) diagnosed in the lower limbs. 1 Compared with proximal DVT (PDVT), IDDVT is often considered a relatively benign disease 2 3 ; however, as many as one-quarter of thrombi in IDDVT extend into the proximal veins and up to one-third are associated with asymptomatic pulmonary embolism (PE) at onset. 4 Moreover, the risk of recurrent venous thromboembolism (VTE) in patients with IDDVT is reported to be between 2 and 4% after approximately 1 year, 2 5 6 and as high as 9 to 25% after approximately 2 to 3 years. 4 7 8 9 Between one-sixth and one-half of recurrent venous thromboembolic events in patients with IDDVT present as PE 2 4 5 6 7 9 10 11 ; however, a meta-analysis suggested that the risk of recurrent VTE presenting as PE is lower in patients with IDDVT than those with PDVT. 12 To date there have been few randomized trials on anticoagulation therapy for IDDVT, and those that have been conducted have yielded conflicting results. 13 14 15

For patients who present with IDDVT, current guidelines do not routinely recommend anticoagulation. 16 In patients with severe symptoms or risk factors for extension (e.g., positive D-dimer, extensive thrombus or close to proximal veins, unprovoked IDDVT, active cancer, or inpatient status), initial treatment with an anticoagulant is suggested. However, in patients without severe symptoms or risk factors for extension, initial serial imaging of the deep veins is suggested to monitor thrombus resolution or extension. If the thrombus extends, anticoagulation is then suggested or recommended (depending on whether the thrombus remains confined to the distal veins or extends to the proximal veins). 16 For patients with IDDVT who receive an anticoagulant, treatment recommendations in guidelines are the same as for patients with PDVT, i.e., at least 3 months of treatment, with the non-vitamin K antagonist (non-VKA) oral anticoagulants recommended over VKAs. 16 Despite these recommendations, in practice many patients with IDDVT are treated for only 4 to 6 weeks. 4 8 9

The EINSTEIN DVT study demonstrated that rivaroxaban was a safe and effective treatment for DVT; however, only patients with PDVT were enrolled. 17 Therefore, data are lacking on outcomes in patients with IDDVT treated with rivaroxaban. Patients with IDDVT and PDVT were included in the noninterventional XALIA phase IV study, which demonstrated the safety and effectiveness of the single-drug approach with rivaroxaban for the treatment of DVT in routine clinical practice. 18 The aims of this analysis were to compare baseline characteristics, management strategies, and clinical outcomes in patients with IDDVT versus those with PDVT with or without concomitant distal DVT enrolled in the XALIA study.

## Materials and Methods

### Study Design, Participants, and Procedures

XALIA was a multicenter, international, prospective, noninterventional study of patients with objectively confirmed DVT. Patients could be included if they were aged ≥18 years with objectively confirmed DVT (which included both PDVT and IDDVT, as well as DVT in other venous beds) and an indication to receive anticoagulation treatment for ≥3 months. After the approval of rivaroxaban for the treatment of PE, the protocol was amended to allow the enrolment of patients with DVT and concomitant PE (but not isolated PE). Patients in the safety analysis received rivaroxaban or standard anticoagulation treatment (initial treatment with unfractionated heparin, low-molecular-weight heparin, or fondaparinux, usually overlapping with and followed by a VKA)—because of the noninterventional nature of the study, treatment, dose, and duration were at the attending physician's discretion. The rivaroxaban cohort included patients who received rivaroxaban alone and those who had received heparin/fondaparinux for a maximum of 48 hours before initiating rivaroxaban treatment, consistent with the approach in the EINSTEIN DVT study. 17 Patients who initially received heparin/fondaparinux for >2 to 14 days or a VKA for 1 to 14 days before switching to rivaroxaban were designated as “early switchers.” These patients were not included in the safety analysis. The study observation period ended 12 months after the date of final patient enrolment; therefore, each patient was followed up for at least 12 months.

Patients with IDDVT or patients with PDVT with or without distal vein (or other locations) involvement and who received treatment with rivaroxaban or standard anticoagulation therapy were included in this analysis. Patients were excluded from this subgroup study if they had concomitant PE (because this was considered a more severe disease and likely to influence the physician's behavior and the rate of VTE recurrence), or if they were “early switchers” (because, similarly, these patients were likely to have comorbidities or risk factors which could also influence the physician's behavior and treatment outcomes).

Further details on the XALIA trial design have been described in the XALIA primary paper. 18

### Outcomes

The primary outcomes in XALIA were major bleeding, recurrent VTE, and all-cause mortality. An adverse event was classified as treatment emergent if it started on or after the day of the first dose of rivaroxaban or standard anticoagulation and within 2 days after the last dose. Major bleeding was defined as any of the following: overt bleeding associated with a fall in hemoglobin of ≥2 g/dL; a transfusion of two or more units of packed red blood cells or whole blood; a critical-site bleeding (intracranial, intraspinal, intraocular, pericardial, intraarticular, intramuscular with compartment syndrome, and retroperitoneal); or fatal bleeding. Recurrent VTE was defined as the new onset of symptoms confirmed by diagnostic testing. Secondary outcomes included health care resource use (admissions to hospital and length of stay).

### Statistical Analysis

A descriptive analysis was conducted comparing the crude incidences for the primary outcomes in patients with IDDVT and PDVT. A multivariable Cox regression analysis was performed to calculate the hazard ratio (HR) and 95% confidence interval (CI) associated with potential predictors of recurrent VTE during treatment and after treatment cessation, after maintaining variables that resulted in at least marginal significance ( *p* < 0.10) in the univariate analysis. Potential confounders in the initial univariate analysis included active cancer at baseline (yes/no); age (<60 years/≥60 years); first available creatinine clearance (CrCl; <50 mL/min, ≥50 to <80 mL/min, ≥80 mL/min, unknown); sex (male/female); fragile (yes/no [patients were defined as fragile if they were >75 years old or had a CrCl <50 mL/min or body weight ≤50 kg]); and provoked VTE (yes/no). VTE was classified as provoked if it was caused by the following transient risk factors: surgery, recent trauma/fracture, postpartum or hospitalization (all <3 months before the index VTE), pregnancy, oral contraceptives, hormone replacement therapy, central venous catheter, postthrombotic syndrome, or immobilization. Cancer was not considered in the definitions of provoked or unprovoked VTE. Treatment assignment (rivaroxaban versus standard anticoagulation) and lower extremity DVT location (IDDVT vs. PDVT) were forced covariates in the multivariate analysis irrespective of marginal significance. A stepwise model was used for each multivariate analysis, with *p* = 0.10 used for keeping variables in the model or adding variables to the model, respectively. Finally, the Cox model for treatment-emergent VTE was adjusted for treatment group and cancer, whereas the Cox model for VTE after treatment cessation was adjusted for CrCl, cancer, and treatment group. Lengths of treatment duration and lengths of hospital stay were described by the median (interquartile range).

## Results

### Baseline Demographics and Clinical Characteristics

**Fig. 1:**
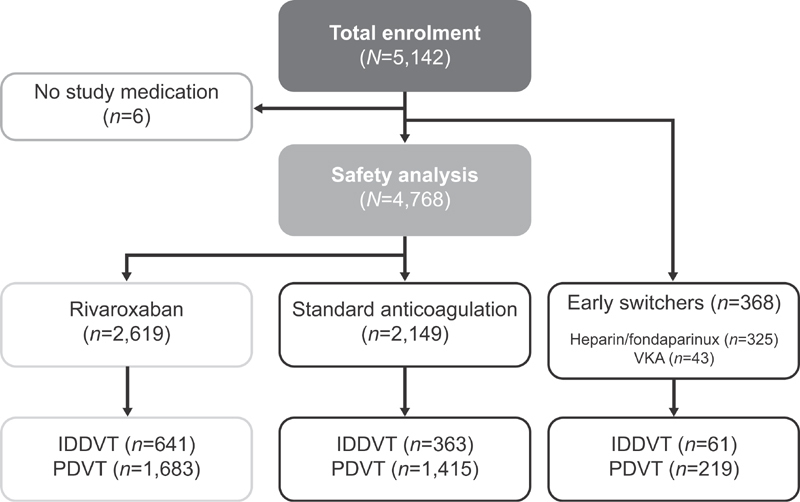
Patient flow through the study. Abbreviations: IDDVT, isolated distal deep-vein thrombosis; PDVT, proximal deep-vein thrombosis; VKA, vitamin K antagonist; VTE, venous thromboembolism.

Between June 26, 2012 and March 31, 2014, 5,142 patients were enrolled in the XALIA study; 6 patients did not take rivaroxaban or standard anticoagulation and were excluded from all subsequent analyses. In the overall XALIA population, 1,065 (20.7%) patients had IDDVT, 3,317 (64.6%) patients had PDVT, 552 (10.7%) patients had DVT with concomitant PE, and 202 (3.9%) patients had VTE in other locations (without PDVT). Sixty-one patients with IDDVT and 219 patients with PDVT were defined as “early switchers,” and were excluded, leaving a total of 4,102 patients in this analysis—1,004 patients with IDDVT and 3,098 with PDVT. Patient flow through the study is shown in Fig. 1 . Baseline characteristics of patients with IDDVT and PDVT are shown in Table 1 . Compared with patients with PDVT, patients with IDDVT were younger, more were female, more had VTE provoked by transient risk factors, and fewer were fragile. Data for patients included in the safety analysis who had IDDVT, subdivided by duration of treatment categorized into ≤90, >90 to 180, and >180 days, are shown in Table 2 .

**Table 1:** Baseline demographics and clinical characteristics of patients with IDDVT and PDVT in the safety analysis set

Characteristic	IDDVT ( *N* = 1,004)	PDVT ( *N* = 3,098)
Age, years, mean ± SD	56.5 ± 17.1	61.0 ± 16.8
Age category, *n* (%)		
<60 y	566 (56.4)	1,315 (42.4)
≥60 y	438 (43.6)	1,783 (57.6)
Male sex, *n* (%)	453 (45.1)	1,704 (55.0)
Weight, kg, mean ± SD	79.6 ± 16.9	82.2 ± 18.3
<50 kg, *n* (%)	13 (1.3)	34 (1.1)
≥50 to 70 kg, *n* (%)	245 (24.4)	643 (20.8)
>70 to <90 kg, *n* (%)	339 (33.8)	1,029 (33.2)
≥90 kg, *n* (%)	200 (19.9)	779 (25.1)
Missing, *n* (%)	207 (20.6)	613 (19.8)
BMI, kg/m ^2^ , mean ± SD	27.6 ± 5.0	28.4 ± 6.3
First available creatinine clearance, *n* (%)		
<30 mL/min	8 (0.8)	57 (1.8)
30 to <50 mL/min	27 (2.7)	173 (5.6)
50 to <80 mL/min	138 (13.7)	557 (18.0)
≥80 mL/min	366 (36.5)	1,253 (40.4)
Missing	465 (46.3)	1,058 (34.2)
Previous VTE, *n* (%)	241 (24.0)	749 (24.2)
Active cancer at baseline, *n* (%)	78 (7.8)	352 (11.4)
Known thrombophilia, *n* (%)	54 (5.4)	177 (5.7)
Previous major bleeding event, *n* (%)	17 (1.7)	70 (2.3)
Hospitalized in previous 3 months, *n* (%)	159 (15.8)	441 (14.2)
Fragile, a *n* (%)	167 (16.6)	753 (24.3)
Provoked VTE, *n* (%)	416 (41.4)	1,053 (34.0)

Abbreviations: BMI, body mass index; IDDVT, isolated distal deep-vein thrombosis; PDVT, proximal deep-vein thrombosis; SD, standard deviation; VTE, venous thromboembolism.

a Fragile: age >75 years or body weight ≤50 kg or creatinine clearance <50 mL/min.

**Table 2:** Baseline demographics and clinical characteristics of patients with IDDVT in the safety analysis set by treatment duration

Characteristic, *n* (%)	≤90 days ( *n* = 272)	>90 to 180 days ( *n* = 452)	>180 days ( *n* = 280)
Age, years			
<60	154 (56.6)	250 (55.3)	162 (57.9)
≥60	118 (43.4)	202 (44.7)	118 (42.1)
Male sex	122 (44.9)	195 (43.1)	136 (48.6)
Region			
Eastern Europe	18 (6.6)	52 (11.5)	50 (17.9)
Western Europe, Canada, and Israel	254 (93.4)	400 (88.5)	230 (81.1)
First available CrCl, mL/min			
<50	11 (4.0)	16 (3.5)	8 (2.9)
≥50 to <80	36 (13.2)	60 (13.3)	42 (15.0)
≥80	99 (36.4)	165 (36.5)	102 (36.4)
Missing	126 (46.3)	211 (46.7)	128 (45.7)
Active cancer at baseline	30 (11.0)	26 (5.8)	22 (7.9)
BMI, kg/m ^2^			
≤25	54 (19.9)	102 (22.6)	52 (18.6)
>25 to ≤35	108 (39.7)	159 (35.2)	102 (36.4)
>35	11 (4.0)	19 (4.2)	23 (8.2)
Missing	99 (36.4)	172 (38.1)	103 (36.8)
Provoked DVT	119 (43.8)	216 (47.8)	81 (28.9)
Fragile a	55 (20.2)	70 (15.5)	42 (15.0)

Abbreviations: BMI, body mass index; CrCl, creatinine clearance; DVT, deep-vein thrombosis; IDDVT, isolated distal deep-vein thrombosis.

a Fragile: age >75 years, body weight ≤50 kg, or CrCl <50 mL/min.

### Treatment Patterns

Details of the treatment patterns are shown in Table 3 . Compared with patients with PDVT, patients with IDDVT were more likely to receive rivaroxaban (641 [63.8%] vs. 1,683 [54.3%] patients, respectively) and were treated for a shorter duration (102 vs. 192 days, respectively). In patients receiving rivaroxaban, a smaller proportion of patients with IDDVT received initial parenteral treatment compared with patients with PDVT (119/641 [18.6%] vs. 493/1,683 [29.3%] patients, respectively). Rivaroxaban was dosed in accordance with the label in most patients with IDDVT and those with PDVT. In patients with IDDVT, 603/641 (94.1%) received an initial rivaroxaban dose of 15 mg twice daily, and 474/641 (73.9%) underwent a planned switch at 21 days to rivaroxaban 20 mg once daily. In patients with PDVT, 1,583/1,683 (94.1%) received an initial rivaroxaban dose of 15 mg twice daily and 1,239/1,683 (73.6%) underwent a planned switch to rivaroxaban 20 mg once daily.

**Table 3:** Treatment details in patients with IDDVT and PDVT in the safety analysis set

	IDDVT ( *N* = 1,004)			PDVT ( *N* = 3,098)		
Anticoagulant, *n* (%)						
Standard anticoagulation	363 (36.2)			1,415 (45.7)		
Heparin/fondaparinux only	107 (10.7)			302 (9.7)		
Heparin/fondaparinux and VKA	256 (25.5)			1,113 (35.9)		
Rivaroxaban	641 (63.8)			1,683 (54.3)		
Initial parenteral treatment for ≤48 h	119 (11.9)			493 (15.9)		
Rivaroxaban only	522 (52.0)			1,190 (38.4)		
	**Overall**	**Standard anticoagulation**	**Rivaroxaban**	**Overall**	**Standard anticoagulation**	**Rivaroxaban**
Treatment duration, median (IQR)	102 (89–188)	107 (88–202)	99 (89–182)	192 (106–366)	207 (109–376)	187 (105–359)
Treatment duration, *n* (%)						
≤90 d	272 (27.1)	100 (27.5)	172 (26.8)	453 (14.6)	223 (15.8)	230 (13.7)
>90 to ≤180 d	452 (45.0)	148 (40.8)	304 (47.4)	779 (25.1)	303 (21.4)	476 (28.3)
>180 d	280 (27.9)	115 (31.7)	165 (25.7)	1,866 (60.2)	889 (62.8)	997 (58.1)
Patients hospitalized for index VTE, *n* (%)	150 (14.9)	92 (25.3)	58 (9.0)	1,107 (35.7)	631 (44.6)	476 (28.3)
Duration of hospital stay, median (IQR)	7 (3–11)	8 (6–13)	4 (2–6)	6 (4–9)	7 (5–10)	5 (3–7)

Abbreviations: IDDVT, isolated distal deep-vein thrombosis; IQR, interquartile range; PDVT, proximal deep-vein thrombosis; VKA, vitamin K antagonist; VTE, venous thromboembolism.

### Treatment-Emergent and Posttreatment Cessation Outcomes

#### Treatment-Emergent Outcomes in Patients with IDDVT versus PDVT

In total, 10 (1.0%) patients with IDDVT and 73 (2.4%) patients with PDVT had a treatment-emergent recurrent venous thromboembolic event. In a multivariate analysis, the risk of recurrent VTE was nonsignificantly lower in patients with IDDVT versus PDVT (adjusted HR: 0.56; 95% CI: 0.29–1.08; *p* = 0.08) ( Table 4 ). Nine (0.9%) patients with IDDVT and 43 (1.4%) patients with PDVT had a treatment-emergent major bleeding event. All-cause mortality occurred in 8 (0.8%) patients with IDDVT and 67 (2.2%) patients with PDVT.

**Table 4:** Adjusted hazard ratios for on-treatment and off-treatment recurrent VTE in patients with IDDVT or PDVT treated with rivaroxaban or standard anticoagulation in safety analysis set

	HR (95% CI)	*p* -Value
** Treatment-emergent recurrent VTE a**		
Rivaroxaban vs. standard anticoagulation	0.64 (0.41–1.01)	0.054
IDDVT vs. PDVT	0.56 (0.29–1.08)	0.084
** Posttreatment cessation recurrent VTE b**		
Rivaroxaban vs. standard anticoagulation	1.17 (0.68–2.04)	0.57
IDDVT vs. PDVT	0.65 (0.32–1.35)	0.25

Abbreviations: CI, confidence interval; DVT, deep-vein thrombosis; HR, hazard ratio; IDDVT, isolated distal vein deep-vein thrombosis; PDVT, proximal deep-vein thrombosis; VTE, venous thromboembolism.

a Model was adjusted for active cancer at baseline (yes/no).

b Model was adjusted for active cancer at baseline (yes/no) and first available CrCl (<50 mL/min, ≥50 to <80 mL/min, ≥80 mL/min, unknown).

#### Outcomes after Treatment Cessation in Patients with IDDVT versus PDVT

Overall, 824 patients with IDDVT (82%) and 2,107 patients with PDVT (68%) continued follow-up after cessation of treatment; the mean follow-up time after treatment cessation was 81 and 104 days, respectively. After treatment cessation, symptomatic recurrent VTE was experienced by 9 (1.1%) patients with IDDVT and 45 (2.1%) patients with PDVT. In a multivariate analysis, the risk of recurrent VTE after treatment cessation was nonsignificantly lower in patients with IDDVT than in those with PDVT (adjusted HR: 0.65; 95% CI: 0.32–1.35; *p* = 0.25) ( Table 4 ). Major bleeding occurred in 3 (0.4%) patients with IDDVT and 7 (0.3%) patients with PDVT. In total, 6 (0.7%) patients with IDDVT and 39 (1.9%) patients with PDVT died in the period between treatment cessation and the end of follow-up.

Additional HRs (stratified by cancer and noncancer only) were calculated, as well as the *p* -values, for interaction between “IDDVT versus PDVT” and “active cancer at baseline (yes/no)” Cox regression analyses for treatment-emergent outcomes and outcomes after treatment cessation. The only significant association was found for the comparison between rivaroxaban and standard anticoagulation in noncancer patients (HR: 0.61; 95% CI: 0.37–0.99; *p* = 0.046). Therefore, there was no statistical sign of an effect of cancer on outcome events in patients with IDDVT (most likely because of the low number of events). Nevertheless, cancer was added as a covariate in the adjusted analysis.

#### Treatment-Emergent Outcomes in Patients with IDDVT or PDVT Treated with Rivaroxaban and Standard Anticoagulation

**Fig. 2:**
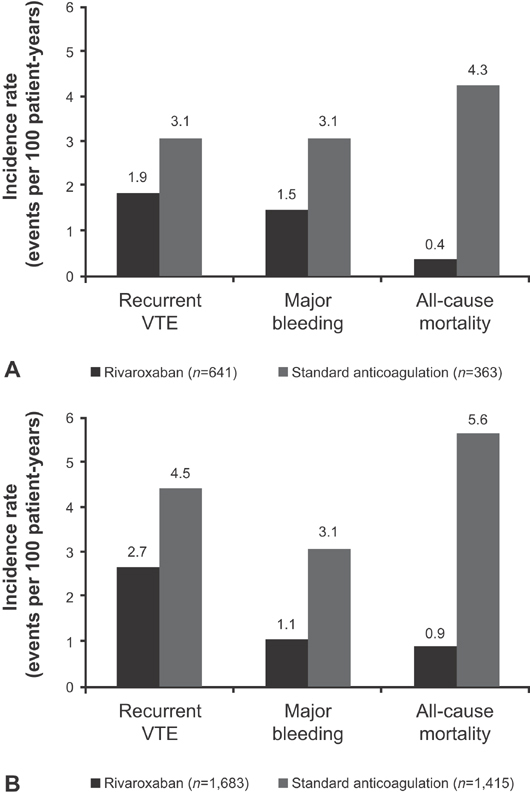
Unadjusted treatment-emergent outcomes in patients with ( **A** ) IDDVT or ( **B** ) PDVT in the safety analysis set treated with rivaroxaban or standard anticoagulation. Abbreviations: IDDVT, isolated distal deep-vein thrombosis; PDVT, proximal deep-vein thrombosis; VTE, venous thromboembolism.

Outcomes from the anticoagulation treatment groups in patients with IDDVT and those with PDVT are reported in Table 5 and illustrated in Fig. 2 . The incidences of recurrent VTE with rivaroxaban and standard anticoagulation were 0.8 and 1.4% in patients with IDDVT and 1.7 and 3.1% in those with PDVT, respectively. Major bleeding incidences with rivaroxaban and standard anticoagulation were 0.6 and 1.4% in patients with IDDVT and 0.7 and 2.2% in those with PDVT, respectively, whereas incidences for all-cause mortality were 0.2 and 1.9%, and 0.6 and 4.0%, respectively.

**Table 5:** Treatment-emergent primary outcomes in patients with IDDVT or PDVT in the safety analysis set treated with standard anticoagulation or rivaroxaban

	IDDVT					PDVT				
	Rivaroxaban ( *n* = 641)		Standard anticoagulation ( *n* = 363)		Risk difference (95% CI)	Rivaroxaban ( *n* = 1,683)		Standard anticoagulation ( *n* = 1,415)		Risk difference (95% CI)
	*n*	%/% per 100 patient-years	*n*	%/% per 100 patient-years		*n*	%/% per 100 patient-years	*n*	%/% per 100 patient-years	
Recurrent VTE	5	0.8/1.9	5	1.4/3.1	−0.68 (−2.67, 0.76)	29	1.7/2.7	44	3.1/4.5	−1.03 (−2.20, 0.04)
Major bleeding	4	0.6/1.5	5	1.4/3.1	−0.27 (−1.98, 0.84)	12	0.7/1.1	31	2.2/3.1	−1.32 (−2.28, −0.49)
All-cause mortality	1	0.2/0.4	7	1.9/4.3	−1.01 (−2.88, 0.12)	10	0.6/0.9	57	4.0/5.6	−1.93 (−3.01, −0.98)

Abbreviations: CI, confidence interval; IDDVT, isolated distal deep-vein thrombosis; PDVT, proximal deep-vein thrombosis; VTE, venous thromboembolism.

Note: Risk differences and 95% CIs were calculated stratified by active cancer at baseline using the Newcombe method.

#### Outcomes after Treatment Cessation in Patients with IDDVT or PDVT Treated with Rivaroxaban and Standard Anticoagulation

Outcomes from the anticoagulation treatment groups in patients with IDDVT and those with PDVT after stopping treatment are reported in Table 6 . The incidences of recurrent VTE with rivaroxaban and standard anticoagulation were 1.3 and 0.7% in patients with IDDVT and 2.0 and 2.3% in patients with PDVT, respectively. Major bleeding incidences with rivaroxaban and standard anticoagulation were 0.2 and 0.7% in patients with IDDVT, respectively, and 0.3% in both groups in patients with PDVT. All-cause mortality with rivaroxaban and standard anticoagulation was 0.6 and 1.0% in the IDDVT group, and 1.5 and 2.3% in the PDVT group, respectively.

**Table 6:** Primary outcomes after treatment cessation in patients with IDDVT or PDVT in patients treated with standard anticoagulation or rivaroxaban who were followed up after treatment cessation

	IDDVT					PDVT				
	Rivaroxaban ( *n* = 526)		Standard anticoagulation ( *n* = 298)		Risk difference (95% CI)	Rivaroxaban ( *n* = 1,241)		Standard anticoagulation ( *n* = 866)		Risk difference (95% CI)
	*n*	%/% per 100 patient-years	*n*	%/% per 100 patient-years		*n*	%/% per 100 patient-years	*n*	%/% per 100 patient-years	
Recurrent VTE	7	1.3/6.5	2	0.7/2.7	0.63 (−1.44, 2.22)	25	2.0/7.6	20	2.3/7.5	−0.15 (−1.53, 1.09)
Major bleeding	1	0.2/0.9	2	0.7/2.7	−0.52 (−2.49, 0.65)	4	0.3/1.2	3	0.3/1.1	−0.06 (−0.85, 0.55)
All-cause mortality	3	0.6/2.8	3	1.0/4.0	−0.08 (−2.04, 1.25)	19	1.5/5.6	20	2.3/7.2	0.03 (−1.12, 0.99)

Abbreviations: CI, confidence interval; IDDVT, isolated distal deep-vein thrombosis; PDVT, proximal deep-vein thrombosis; VTE, venous thromboembolism.

Note: Risk differences and 95% CIs were calculated stratified by active cancer at baseline using the Newcombe method.

## Discussion

The results of this analysis of patients enrolled in the XALIA study confirm differences in baseline characteristics between patients with IDDVT and those with PDVT. This finding is consistent with data from the RIETE registry and the OPTIMEV and DOTAVK studies, in which patients with IDDVT were younger and less likely to have unprovoked VTE than patients with PDVT. 5 6 10 Reported also in previous studies, patients with IDDVT were treated with anticoagulants for a shorter period of time than patients with PDVT. 2 5 6 These differences in treatment duration may reflect the perception of IDDVT as a more benign disease than PDVT, but may also be driven by the differences in clinical characteristics and underlying risk factors between patients with IDDVT and those with PDVT.

The incidences of recurrent VTE during treatment and after treatment cessation were numerically lower in patients with IDDVT than in those with PDVT, although this difference was nonstatistically significant after adjustment for differences in baseline characteristics. This finding is also consistent with results from an analysis of patients with IDDVT or PDVT in the RIETE registry 10 and apparently supports the recommendations in the guidelines from the American College of Chest Physicians (ACCP) that PDVT and IDDVT should be treated for the same length of time. 16 Other studies have reported lower crude incidences of VTE recurrence in patients with IDDVT compared with those with PDVT. In the prospective DOTAVK and OPTIMEV studies and a retrospective single-center cohort study, the incidences of recurrent VTE in patients with IDDVT were approximately half of those seen in patients with PDVT; however, with the exception of the study by Barco et al, no adjustments were made for differences in baseline characteristics. 2 5 6 There have been few randomized trials to date on anticoagulation therapy for IDDVT and results have been conflicting. In a 1985 study comparing 3 months' warfarin treatment versus no treatment for calf vein thrombosis, none of the patients who received warfarin experienced a recurrent venous thromboembolic event, whereas the event rate in the no-treatment group was 29% 13 ; however, the study was small (51 patients in total) and included patients at high risk of a recurrent event (based on symptomatic painful thrombosis at enrolment and high pain scores, which were associated with recurrent events). A subsequent study in 2010 compared the safety and efficacy of short-term (10 days) low-molecular-weight heparin plus compression therapy versus compression therapy alone for isolated calf muscle DVT; efficacy was not superior with anticoagulation, although there were no major bleeding events in either group. 14 In a 2016 study of nadroparin versus placebo for 42 days for the treatment of calf vein DVT in low-risk outpatients, 15 nadroparin was not superior to placebo for reducing the risk of proximal extension or recurrent VTE and was associated with a higher rate of bleeding.

Based on the results of this and previous studies, the identification of patients with IDDVT requiring the same treatment intensity and duration as for patients with PDVT remains unclear. Although the differences in the incidence of recurrent events in our study were not statistically significant, patients enrolled in XALIA were required to have an indication to receive 3 months' anticoagulation to be eligible. This means that IDDVT patients with more risk factors for VTE recurrence were more likely to have been enrolled than those with fewer risk factors; therefore, it is uncertain whether the outcomes observed in this analysis are representative of the wider IDDVT patient population. In addition, outcomes with rivaroxaban and standard anticoagulation in the on-treatment phase may have been influenced by a greater imbalance in baseline risk factors and comorbidities in the rivaroxaban versus standard anticoagulation group in patients with PDVT compared with those with IDDVT, or because a perception of IDDVT as being more benign than PDVT impacted prescribing decisions (which is possibly reflected in the lower rates of standard anticoagulation prescribing in the IDDVT group). Finally, treatment duration in patients with PDVT was longer than in patients with IDDVT, which may have impacted between-treatment differences in each group. Because of these reasons, no conclusions can be drawn regarding the differences in incidence rates between rivaroxaban and standard anticoagulation users.

## Limitations

XALIA was a noninterventional study, and treatment decisions were at the physician's discretion with potential for selection bias. In particular, as mentioned above, because patients were only eligible for inclusion if the intended duration of treatment was 3 months or more, 19 lower-risk patients with IDDVT that some physicians would normally treat for a shorter period would have been ineligible for inclusion. For example, this is the case with a thrombus confined to the muscular veins (i.e., soleus/gastrocnemius veins), which is considered to have a lower risk of extension than a thrombus confined to the axial veins (i.e., true deep, peroneal, or tibial veins). 16 Unfortunately, information on the site of distal DVT was not collected. In addition, XALIA was an open-label study, which raised the possibility of bias in the investigator reporting of events. However, this was addressed by the use of objective diagnostic methods and by the adjudication of all events by the Adjudication Committee, which was blinded to treatment choice. Because of the low event numbers, full control of confounding factors was not possible in the multivariate analysis and hence the results may still have been influenced by differences in demographics. Unlike in the XALIA primary analysis, a propensity score-adjusted analysis was not possible in this sub-study because of the small group sizes. Furthermore, independent risk factors may have been masked by the effect of treatment group comparison and thus would not have appeared in the stepwise regression analysis. Finally, the comparison of events occurring after cessation of treatment may have been influenced by the different proportions of patients being followed up.

## Conclusions

Patients with IDDVT had fewer comorbidities and were more frequently treated with rivaroxaban than those with PDVT. This may reflect IDDVT being considered more benign than PDVT. Consequently, the risks of VTE recurrence in patients with IDDVT treated with either rivaroxaban or standard anticoagulation were lower than in patients with PDVT, although this difference was not statistically significant after adjustments for some major underlying factors. Uncertainty still exists as to whether patients with IDDVT require anticoagulation and, if so, if they require the same treatment intensity and duration as for patients with PDVT. Indeed, the study population of XALIA did not include patients with IDDVT who received less than 3 months' anticoagulation therapy and would likely have fewer risk factors and a lower risk for recurrence than those eligible to enroll. Therefore, additional studies specifically designed to address this issue are warranted.
